# Influence of Sintering Strategy on the Characteristics of Sol-Gel Ba_1−*x*_Ce*_x_*Ti_1−*x*/4_O_3_ Ceramics

**DOI:** 10.3390/nano9121675

**Published:** 2019-11-23

**Authors:** Cătălina A. Stanciu, Ioana Pintilie, Adrian Surdu, Roxana Truşcă, Bogdan S. Vasile, Mihai Eftimie, Adelina C. Ianculescu

**Affiliations:** 1 Department of Science and Enginnering of Oxide Materials and Nanomaterials, University POLITEHNICA of Bucharest, Bucharest 060042, Romania; catalina.a.stanciu@gmail.com (C.A.S.); truscaroxana@yahoo.com (R.T.); bogdan.vasile@upb.ro (B.S.V.);; 2 National Institute for Lasers, Plasma and Radiation Physics, P.O. Box MG54, Bucharest-Magurele 077125, Romania; 3 National Institute of Materials Physics, P.O. Box MG-7, Bucharest-Magurele 077125, Romania; ioana@infim.ro

**Keywords:** sol-gel, Ce^3+^-doped BaTiO_3_, dielectric permittivity, ferroelectric–paraelectric phase transition, spark plasma sintering

## Abstract

Single-phase Ce^3+^-doped BaTiO_3_ powders described by the nominal formula Ba_1−*x*_Ce*_x_*Ti_1−*x*/4_O_3_ with *x* = 0.005 and 0.05 were synthesized by the acetate variant of the sol-gel method. The structural parameters, particle size, and morphology are strongly dependent on the Ce^3+^ content. From these powders, dense ceramics were prepared by conventional sintering at 1300 °C for 2 h, as well as by spark plasma sintering at 1050 °C for 2 min. For the conventionally sintered ceramics, the XRD data and the dielectric and hysteresis measurements reveal that at room temperature, the specimen with low cerium content (*x* = 0.005) was in the ferroelectric state, while the samples with significantly higher Ce^3+^ concentration (*x* = 0.05) were found to be in the proximity of the ferroelectric–paraelectric phase transition. The sample with low solute content after spark plasma sintering exhibited insulating behavior, with significantly higher values of relative permittivity and dielectric losses over the entire investigated temperature range relative to the conventionally sintered sample of similar composition. The spark-plasma-sintered Ce-BaTiO_3_ specimen with high solute content (*x* = 0.05) showed a fine-grained microstructure and an almost temperature-independent colossal dielectric constant which originated from very high interfacial polarization.

## 1. Introduction

Due to their environmentally friendly character, along with multiple unique and useful properties such as high dielectric permittivity, piezoelectric, pyroelectric and ferroelectric behavior, and PTCR (positive temperature coefficient of resistivity) effect and high endurance under DC field stress, BaTiO_3_-based ceramics are widely used in microelectronics applications as multilayer ceramic capacitors (MLCC), ferroelectric memory (FeRAM), energy harvester actuators, transducers, IR sensors, thermistors, controllers, and phase shifters [1,2,3,4,5].

Taking into account the flexibility of the perovskite lattice, a proper approach to tailoring the functional behavior of BaTiO_3_-based ceramics consists of adopting an adequate compositional design, which involves doping strategies or the formation of solid solutions.

It is well known that *A*-site donor doping in BaTiO_3_ involving the incorporation of larger rare-earth ions such as La^3+^ and Nd^3+^ [6,7,8,9,10,11,12] in the perovskite lattice is more effective in shifting the Curie temperature toward lower values than the effect induced by homovalent species such as Sr^2+^ and Ca^2+^ [13,14,15,16,17]. The content of donor dopant also exhibits a complicated influence on the electrical properties. Thus, a higher content of donor solutes, exceeding a critical composition of ~0.5 atom %, but found within their solubility range, determines a typical insulating behavior determined by a compensating mechanism via cation vacancies, in order to maintain electroneutrality [18]. For concentrations below the already mentioned critical value, such donor-doped BaTiO_3_ systems behave at room temperature like semiconductors, with a well-marked PTCR effect just above the Curie temperature due to the compensation of the donor dopant’s positive “extra-charge” inside the ceramic grains via electrons [19]. However, a previous study revealed that even in the case of lightly La-doped BaTiO_3_ ceramics, changes in the defect chemistry by modifying the preparation and sintering strategy can induce a typical insulating, nonlinear behavior at room temperature [9].

Because of its variable oxidation state, cerium can be incorporated as Ce^3+^ on Ba sites, acting as a donor dopant [20,21,22]; as Ce^4+^ on Ti^4+^, forming homovalent solid solutions [23,24,25,26]; or simultaneously as Ce^3+^ on *A* sites and Ce^4+^ on *B* sites of the *AB*O_3_ perovskite lattice [27,28]. The valence of cerium and, consequently, its site occupancy and solubility in the host BaTiO_3_ lattice are strongly dependent on the starting Ba/Ti ratio and oxygen partial pressure [28].

According to Equation (1), “built-in” titanium vacancies as compensating defects for the effective positive extra-charge of the donor dopant have to be stipulated in the starting formula, and sintering in air at temperatures below 1350 °C followed by slow cooling has to be carried out in order to obtain single-phase, insulating ceramics [6,7,8,29,30].

(1) $$4{\mathrm{CeO}}_{2}+3{\mathrm{TiO}}_{2}\to 4{\mathrm{Ce}}_{\mathrm{Ba}}^{•}+3{\mathrm{Ti}}_{\mathrm{Ti}}^{\times }+V_{\mathrm{Ti}}^{\prime \prime \prime \prime }+12O_{O}^{\times }+O_{2}(g)$$

In the present work, we considered two compositions, corresponding to the nominal formula Ba_1−x_Ce_x_Ti_1−x/4_O_3_, with *x* = 0.005 and 0.05, respectively. As already mentioned, a Ce^3+^ doping level of 0.5 atom % (*x* = 0.005) is considered the “critical” threshold for which a semiconductor–insulator transition accompanied by the so-called “grain growth anomaly” takes place, while a one-order-of-magnitude-higher solute concentration of 5 atom % (*x* = 0.05) represents the “morphotropic” concentration reported in the literature for shifting the Curie temperature in the proximity of room temperature [20,21,22].

Various techniques such as the classical solid-state reaction method [20,31,32], the sol-gel route [33], and the Pechini procedure [21,22,34,35] have been reported for the preparation of Ce^3+^-doped BaTiO_3_ ceramics. The “acetate” variant of the sol-gel route is known as an excellent preparation technique for controlling the stoichiometry and microstructure homogeneity of doped BaTiO_3_-based products [33,36,37,38], so we considered it as adequate for our study. The sol-gel powders were labelled after their composition as BCT-005 and BCT-05, respectively.

In the last several years, spark plasma sintering has quite often been used as an alternative method to the classical sintering procedure in order to obtain highly densified, nanocrystalline ceramics from powders prepared by various wet chemical methods [39,40,41,42]. BaTiO_3_ ceramic bodies with minimum grain growth and therefore exhibiting grain size (GS) down to 30 nm and relative density of 95–99% have been produced by this procedure [43,44]. Unlike pure BaTiO_3_, spark-plasma-sintered ceramics derived from substituted barium titanate solid solutions are much less studied from the point of view of their microstructure and electrical characteristics [45,46,47,48]. Regarding the Ce^3+^-doped BaTiO_3_ system, only results describing the characteristics of the ceramics consolidated by conventional sintering are reported in the literature. No data referring to the microstructure and electrical behavior of cerium-doped barium titanate ceramics obtained by field-assisted sintering techniques were found. Therefore, in order to obtain fine-grained ceramics and to investigate the influence of grain size on the electrical behavior, the sol-gel Ce^3+^-doped BaTiO_3_ powders were also consolidated by spark plasma sintering. The structural parameters, microstructure, and functional properties of the resulting ceramics after conventional sintering and spark plasma sintering are comparatively discussed. The ceramic specimens were differentiated after the sintering procedure by adding to the powder labels the indicative “CS” for conventional sintering and “SPS” for spark plasma sintering.

## 2. Materials and Methods

### 2.1. Sample Preparation

Barium acetate (Ba(CH_3_COO)_2_, 99%, Sigma Aldrich, Saint Louis, MO, USA), titanium (IV) isopropoxide 97% solution in 2-propanol (Ti[OCH(CH_3_)_2_]_4_, Sigma Aldrich, Saint Louis, MO, USA), and cerium acetate (Ce(CH_3_CO_2_)_3_, 99.9%, Sigma Aldrich, Saint Louis, MO, USA) were used as starting reagents. Two different solutions were prepared by dissolving appropriate amounts of barium acetate in acetic acid and cerium acetate in acetic acid, at 70 °C, under continuous stirring. As stabilizers for the sol, 2-methoxyethanol and acetylacetone in a 2:1 volume ratio were used. Another solution was formed by mixing titanium isopropoxide in 2-propanol. The barium acetate solution was added to the titanium isopropoxide solution under continuous stirring. Then, the cerium acetate solution was added to the barium and titanium mixture solution. In a subsequent step, acetylacetone (CH_3_COCH_2_COCH_3_, 97%, Aldrich, Saint Louis, MO, USA) was added to the as-obtained solution. The resulting clear, yellowish sols were then heated on a hotplate under magnetic stirring at 80 °C for 2 h. During this process, the solutions became increasingly viscous, until yellow-brown gels were obtained. These gels were thermally treated at 100 °C for 12 h, resulting in brown-orange glassy resins which were lightly ground in a mortar. The amorphous precursor powders were then thermally treated in static air at 900 °C for 2 h in a muffle furnace, using a heating rate of 5 °C min^−1^. An intermediate plateau of 2 h at a lower temperature of 450 °C was also carried out in order to ensure the complete removal of organic material.

The as-synthesized oxide powders with compositions described by the nominal formula Ba_1−x_Ce_x_Ti_1−x/4_O_3_ (*x* = 0.005 and 0.05) were milled and shaped by uniaxial die pressing at 174 MPa into pellets with a diameter of ~13 mm and a thickness of ~1.2–1.6 mm, using a small amount of organic binder (5% PVA aqueous solution). The green bodies were sintered in a muffle furnace in static air at 1300 °C for a 4 h plateau, using a heating rate of 5 °C·min^−1^, and then they were slowly cooled (at the normal cooling rate of the furnace) to room temperature in order to obtain dense Ce^3+^-doped BaTiO_3_ ceramic samples. After sintering, the color of the ceramic samples varied from light orange to reddish with varying Ce^3+^ content from 0.5 atom % to 5 atom %.

Amounts of sol-gel powders with the abovementioned compositions were also used to prepare dense ceramics by spark plasma sintering (SPS). The powders were poured into a graphite die and then sintered under vacuum to create dense ceramic pellets of 10 mm diameter and ~1 mm thickness, using commercial SPS equipment (FCT Systeme GmbH, Rauenstein, Germany, Spark Plasma Sintering Furnace type HP D 1.25). The temperature was raised at a fixed heating rate of 100 °C·min^−1^ under a constant applied pressure of 50 MPa and then held at a constant temperature of 1050 °C for 2 min. Rapid heating was provided by a pulsed DC current. After polishing, all the ceramic samples were annealed in air at 1000 °C for 16 h, with a heating rate of 10 °C min^−1^. The aims of this post-sintering thermal treatment were (*i*) to reduce the concentration of oxygen vacancies originating from the reducing conditions of the SPS process and to ensure the re-oxidation of the Ti^3+^ species to Ti^4+^; (*ii*) to remove possible surface carbon contamination; and (*iii*) to relieve the residual stresses arising either from the SPS process or from polishing.

### 2.2. Sample Characterization

To monitor the decomposition process of the precursors, thermal analysis investigations were performed in a static air atmosphere up to 1200 °C, with a heating rate of 10 °C min^−1^, by using a NETZSCH STA 409 PC LUXX (Selb, Germany) thermal analyzer.

The phase purity and crystal structure of the Ce^3+^-doped BaTiO_3_ powders and related ceramics were determined by X-ray diffraction (XRD) investigations, performed at room temperature (23 °C) by means of a SHIMADZU XRD 6000 diffractometer (Kyoto, Japan), using Ni-filtered Cu Kα radiation (λ = 1.5406 Å). The measurements were performed in *θ*–2*θ* mode with a scan step increment of 0.02° and a counting time of 1 s/step in the 2*θ* range of (20°–80°). Phase identification was performed using HighScore Plus 3.0e software (PANalytical, Almelo, The Netherlands), PANalytical, Almelo, The Netherlands connected to the ICDD PDF-4+ 2017 database. To estimate the structural characteristics, the same step increment but with a counting time of 10 s/step in the same 2*θ* range was used. Lattice parameters were refined by the Rietveld method. After removing the instrumental contribution, the full width at half-maximum (FWHM) of the diffraction peaks can be interpreted in terms of the crystallite size and lattice strain. A pseudo-Voigt function was used to refine the shapes of the Ce^3+^-doped BaTiO_3_ peaks.

For a high-accuracy estimation of the morphology and crystallinity degree of the constitutive Ce^3+^-doped BaTiO_3_ particles, transmission electron microscopy (TEM/HRTEM) and selected-area electron diffraction (SAED) investigations were performed. The bright-field and high-resolution images were collected by using a TecnaiTM G^2^ F30 S-TWIN transmission electron microscope (FEI Co., Hillsboro, OR, USA). For these purposes, small amounts of powdered samples were suspended in ethanol by 15 min ultrasonication. A drop of suspension was put onto a 400 mesh, holey carbon-coated film Cu grid and dried. The average particle size for each Ce^3+^-doped BaTiO_3_ powder was calculated using Origin Pro 9.0 software (OriginLab, Northampton, MA, USA) by taking into account size measurements on ~60 particles (from images of appropriate magnifications obtained from various microscopic fields) performed using the microscope software Digital Micrograph 1.8.0 (Gatan, Sarasota, FL, USA).

A high-resolution FEI QUANTA INSPECT F microscope with field emission gun (FEI Co., Hillsboro, OR, USA), coupled with energy-dispersive X-ray spectroscopy (EDX), was used to analyze the microstructure and the elemental composition of the ceramics obtained by both conventional sintering and spark plasma sintering. The grain size of the ceramics was determined as the mean intercept length by taking into account measurements on ~70–80 grains. The relative density values of the sintered ceramic pellets were roughly determined as the ratio between the apparent density measured by Archimedes’ principle and their crystallographic (theoretical) density calculated from the diffraction data.

The dielectric behavior was studied by performing electrical measurements of capacitance and dielectric losses, with the aid of an HP 4194A impedance/gain analyzer, on samples having a parallel-plate capacitor configuration (by applying Ag–Ag electrodes on both polished surfaces of the sintered ceramic disks of about 1 mm thickness). The measurements were performed in a Janis cryostat, in a temperature range of −250 to +200 °C, and at different fixed frequencies of the small-amplitude AC signal, in the range of 10^3^–10^5^ Hz. All these measurements were performed in vacuum, during heating up, with a heating rate of β = 0.01 K/s (0.6 K/minute). It is thought that these measurement conditions ensure a good thermal equilibrium for the sample during the capacitance measurements.

Hysteresis measurements, revealing the total (dynamic) polarization versus the electric field, were performed at ambient temperature (22 °C) using a “Premier II” ferritester together with the “Vision” software (Radiant Technologies, Inc., Albuquerque, NM, USA, version 4.2.0) package provided by Radiant Technologies Inc (Radiant Technologies, Inc., Albuquerque, NM, USA). Measurements were performed using a 1 s pulse (1 Hz measurement frequency).

## 3. Results and Discussion

### 3.1. Thermal Behaviour of Precursors

**Figure 1:**
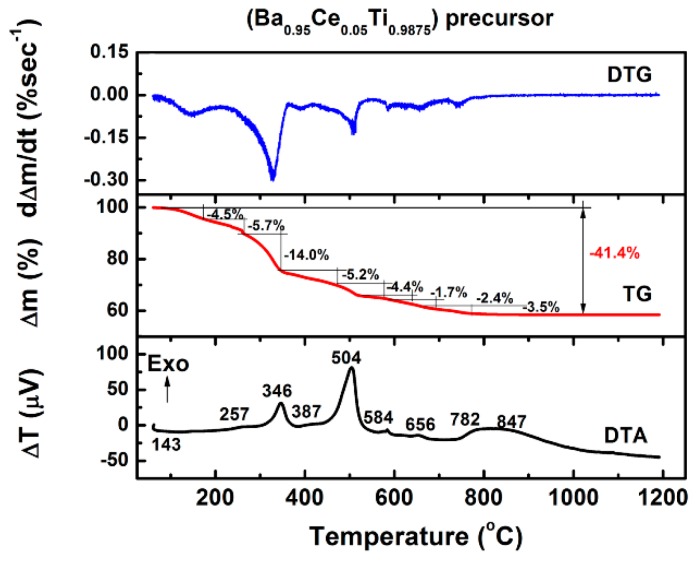
Thermal analysis curves of the precursor powder with the highest cerium content (x = 0.05).

Thermal analysis methods allowed us to investigate the transformation of the amorphous precursors into oxide powders during heating. Monitoring the thermal behavior of the gel precursor with the highest cerium content showed that the decomposition process is complex, involving three main decomposition stages showed on the derivative thermogravimetry (DTG) curve in the temperature ranges 100–250 °C, 250–540 °C, and 540–785 °C, respectively. Each of these main stages consists of several decomposition steps indicated by corresponding thermal effects recorded on the differential thermal analysis (DTA) curve and accompanied by mass losses observed on the thermogravimetric (TG) curve, as shown for the gel precursor with the highest cerium content in Figure 1.

In the low temperature range, the DTA curve exhibited a weak endothermic effect with a maximum centered around ~143 °C and accompanied by a slight mass loss of 4.5% on the TG curve, which was attributed to the release of adsorbed water and to the evaporation of the solvents. At temperatures ranging between 250 °C and 540 °C, combustion of the organic groups took place in four different steps as indicated by the exothermic effects whose maxima are located at 257, 346, 387, and 504 °C, respectively. For these processes, a cumulative mass loss of 24.9% was estimated from the thermogravimetric measurements. Further, a succession of weak, exothermic effects was recorded in the temperature range of 540–785 °C, with the maxima centered at 584 °C, 656 °C, and 782 °C on the DTA curve. These effects might appear to result from two opposite processes, i.e., endothermic decomposition of some intermediate carbonated phases such as BaCO_3_ and Ba_2_Ti_2_O_5_·CO_3_ due to combustion, accompanied by a mass loss of 12%, occurring simultaneously with the exothermic formation of the perovskite skeleton, which seems to prevail. The last decomposition step (~782 °C) is clearly overlapped with the formation of the perovskite Ce-BaTiO_3_ phase by a complex process involving crystallization and/or solid state reaction between small amounts of barium- and titanium-rich secondary phases (Ba_2_TiO_4_ and BaTi_2_O_5_), which usually originate from some residual chemical heterogeneities in the precursor gels. This overlapping results in a broad, flattened, high-temperature feature on the DTA curve. The formation/crystallization of the Ce-BaTiO_3_ solid solution was completed at ~950 °C.

### 3.2. Ce^3+^-Doped BaTiO_3_ Powders

#### 3.2.1. Phase Composition and Crystalline Structure

**Figure 2:**
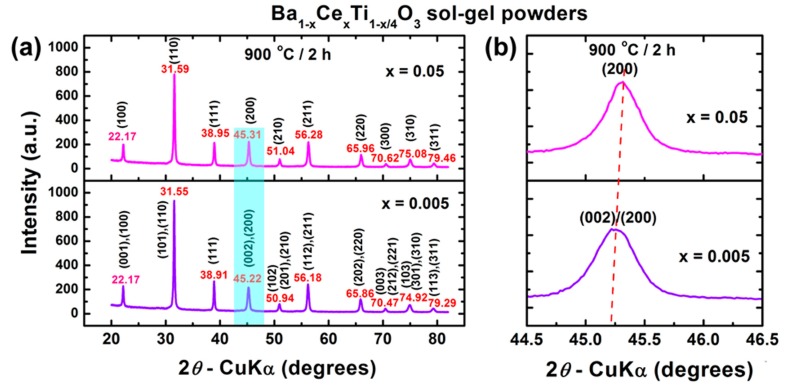
(**a**) XRD patterns recorded at room temperature for the 0.5% and 5% Ce^3+^-doped BaTiO_3_ powders thermally treated at 900 °C /2 h, and (**b**) details (light blue rectangle of Figure 2a) of the region corresponding to diffraction angles 2*θ* = 44.5°–46.5°.

The X-ray diffraction patterns recorded at room temperature for the oxide powders obtained after annealing at 900 °C for 2 h show the presence of well-crystallized Ce-BaTiO_3_ perovskite as a unique phase, irrespective of cerium content (Figure 2a).

The analysis of the positions and profiles of the main diffraction peaks revealed the presence of two features: (*i*) a shift in the main reflections toward higher diffraction angles with increasing Ce^3+^ content (Figure 2a), induced by the difference in the ionic radius values of the *A*-site substituting and substituted species (*r*(Ce^3+^) = 1.34 Å compared to *r*(Ba^2+^) = 1.61 Å [49]), proving the incorporation of the solute on the barium site; and (*ii*) the absence of splitting of the (200) reflection in the XRD patterns of the powders under investigation, which seems to indicate a cubic structure (Figure 2b).

However, Rietveld refinement revealed that only the heavily doped powder (x = 0.05) exhibited cubic Pm3m symmetry of the unit cell (ICDD card no. 01-083-3859). For the powder with lower Ce^3+^ addition (x = 0.005), the best fit for the corresponding XRD pattern (the lowest values of the Rietveld parameters *R*_p_, *R_wp_*, and χ^2^) was obtained by using the P4mm space group, specific to the tetragonal structure (ICDD card no. 01-081-8524). However, the low *c*/*a* ratio suggests that the size of the particles is small enough to induce internal strains which seem to affect tetragonality in the Ba_0.995_Ce_0.005_Ti_0.99875_O_3_ powder. The stabilization of the cubic structure in the heavily doped Ba_0.95_Ce_0.05_Ti_0.9875_O_3_ powder is determined by several factors: (*i*) a significant increase in the concentration of smaller Ce^3+^ ions on barium sites; (*ii*) a consequent increase in the concentration of titanium vacancies [35]; and (*iii*) an increase of the internal strains simultaneous to the decrease in crystallite size induced by the greater addition of donor dopant.

The obvious decrease in the lattice parameters and the contraction of the unit cell volume are also related to the increasing cerium content incorporated on barium sites, taking into account the lower ionic radius of Ce^3+^ with respect to Ba^2+^, as mentioned above. The structural parameters of the perovskite phases are presented in Table 1.

#### 3.2.2. Morphology

**Figure 3:**
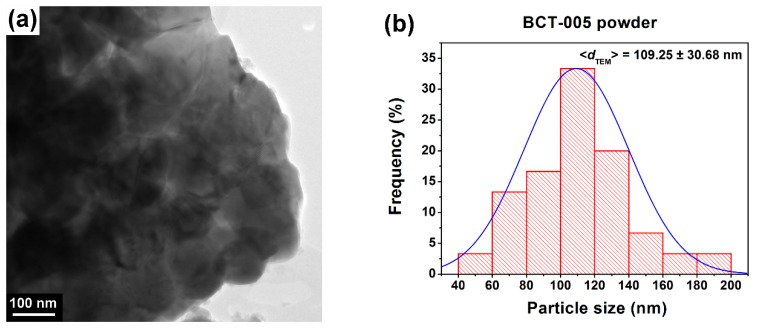

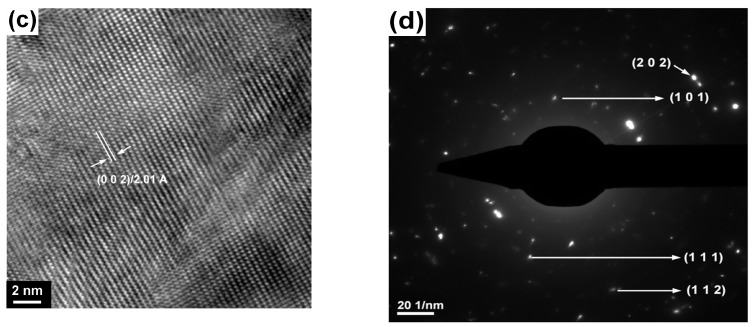
(**a**) TEM image, (**b**) histogram indicating the particle size distribution, (**c**) HRTEM image, and (**d**) selected-area electron diffraction (SAED) pattern of the sol-gel Ba_0.995_Ce_0.005_Ti_0.99875_O_3_ (BCT-005) powder.

**Figure 4:**
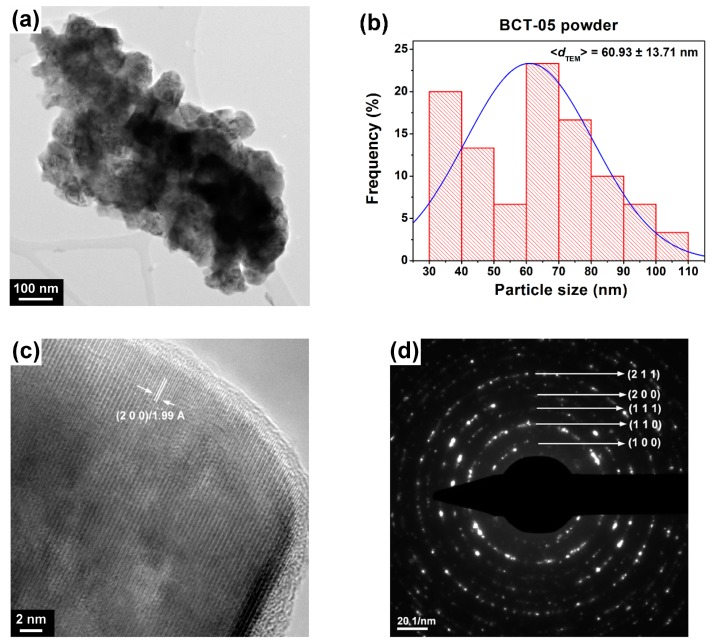
(**a**) TEM image, (**b**) histogram indicating the particle size distribution, (**c**) HRTEM image, and (**d**) SAED pattern of the sol-gel Ba_0.95_Ce_0.05_Ti_0.9875_O_3_ (BCT-05) powder.

TEM/HRTEM analyses coupled with SAED investigations revealed that the Ce^3+^-doped BaTiO_3_ powders consist of well-crystallized, polyhedral particles with well-defined edges and rounded corners (Figure 3a–d and Figure 4a–c). A high agglomeration tendency was observed for the particles of the sample BCT-05, while partially sintered blocks were noticed in the case of the powder BCT-005. Average particle sizes of 109.25 nm and 60.93 nm were estimated for the powders BCT-005 and BCT-05, respectively (Figure 3b and Figure 4b, Table 1). In the analysis of the influence of Ce^3+^ content on the powder morphology, decreases in both the average particle size and the aggregation tendency when the dopant content increased from 0.5 atom % to 5 atom % were indicated by the TEM images of Figure 3a and Figure 4a. When comparing the values of the average crystallite size calculated from the diffraction data with the values corresponding to the average particle size estimated from TEM analysis, one can assume that in both cases most of the particles are not single crystals. Thus, the grains inside the aggregates observed in the powdered sample BCT-005 seem to consist of ~2–5 crystallites (Figure 3b). A more complicated particle size distribution was determined for the powdered sample BCT-05, in which a major proportion of the polycrystalline particles (consisting of ~2–4 crystallites) coexists with a smaller fraction of single-crystal particles (Figure 4b).

The HRTEM images in Figure 3c and Figure 4c clearly show long-range highly ordered fringes spaced at 2.01 and 1.99 Å, corresponding to the (002) and (200) crystalline planes of the tetragonal and cubic structures of the powdered samples BCT-005 and BCT-05, respectively. The high crystallinity degree of the randomly oriented particles/grains was also indicated by the bright spots, forming well-defined diffraction rings assigned to several crystalline planes of the perovskite phase in the SAED patterns in Figure 3d and Figure 4d. The results of the SAED investigations are in good agreement with the XRD data, indicating the tetragonal distortion of the specimen BCT-005 and cubic symmetry of the unit cell for the powder BCT-05.

### 3.3. Ce^3+^-Doped BaTiO_3_ Ceramics

#### 3.3.1. Phase Composition and Crystalline Structure

**Figure 5:**
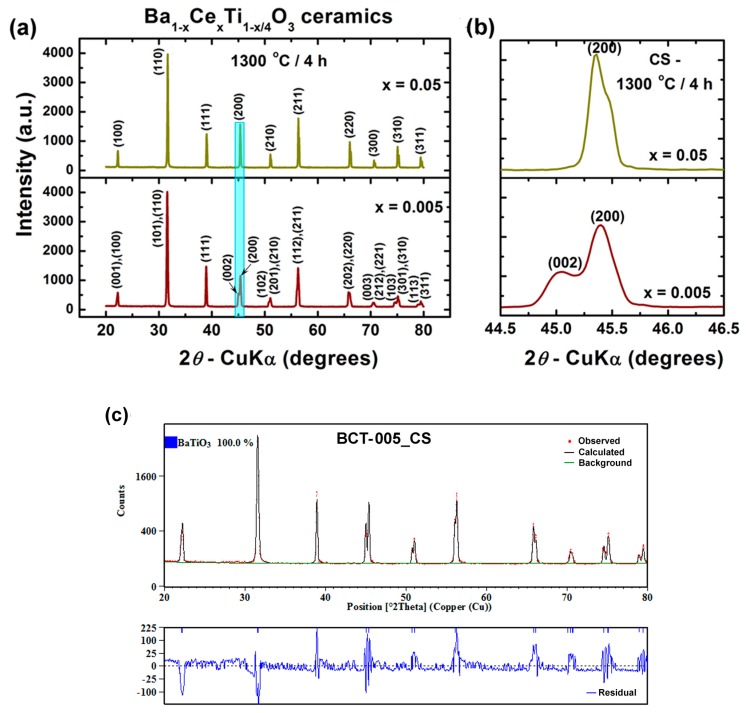

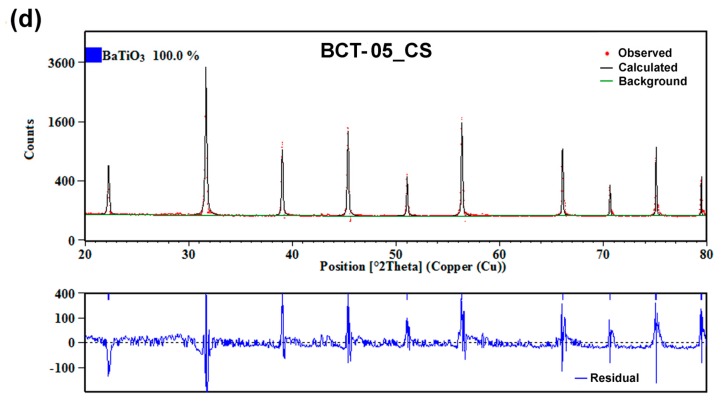
(**a**) XRD patterns recorded at room temperature for Ba_1−x_Ce_x_Ti_1−x/4_O_3_ ceramics obtained by conventional sintering (CS) at 1300 °C/4 h; (**b**) details (light blue rectangle of Figure 5a) of the region corresponding to diffraction angles 2*θ* = 44.5°–46.5°; (**c**,**d**) results of Rietveld analyses of X-ray diffraction data for the conventionally sintered ceramics: (**c**) BCT-005_CS and (**d**) BCT-05_CS.

The XRD patterns of the ceramics derived from sol-gel powders and conventionally sintered at 1300 °C/4 h show, irrespective of the cerium content, the obtaining of single-phase compositions consisting of well-crystallized Ce^3+^-BaTiO_3_ solid solutions as identified by the main reflections of the perovskite structure (Figure 5a). These observations were also sustained by the results obtained from the Rietveld analysis, which indicated the presence of 100% Ce^3+^-BaTiO_3_ in both ceramics BCT-005_CS and BCT-05_CS (Table 2 and Figure 5c,d). As in the case of the starting powders, the formation of single-phase ceramics, free of any barium- or titanium-rich secondary phases, suggests the effectiveness of the “built-in” tetra-ionized titanium vacancies in compensating the supplementary positive electrical charge induced by the presence of the Ce^3+^ solute on Ba sites.

From a structural point of view, unlike the starting oxide powder, in the case of the ceramic sample doped with 0.5 atom % Ce^3+^ (BCT-005_CS), the splitting of the (200) reflection in two adjacent (002) and (200) peaks is more significant, indicating higher tetragonality of the unit cell due to the microstructure coarsening induced by sintering (Figure 5b). For the BaTiO_3_ specimen with a higher cerium concentration (5 atom %), even though the two (002) and (200) peaks merge into a single symmetric (200) peak, the profile of this peak remains complicated (Figure 5b). This seems to suggest the presence of a mixture of tetragonal and cubic modifications, which means that, for the BCT-05_CS specimen, ferroelectric–paraelectric phase transition might occur near room temperature.

Calculation of the structural parameters based on the XRD data sustained this assertion, showing a typical tetragonal structure for the lightly doped sample BCT005_CS, while a cubic structure was determined for the specimen BCT05_CS (Table 2).

**Figure 6:**
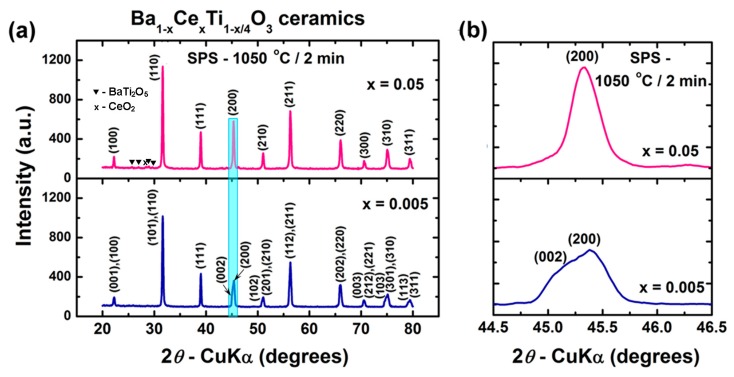

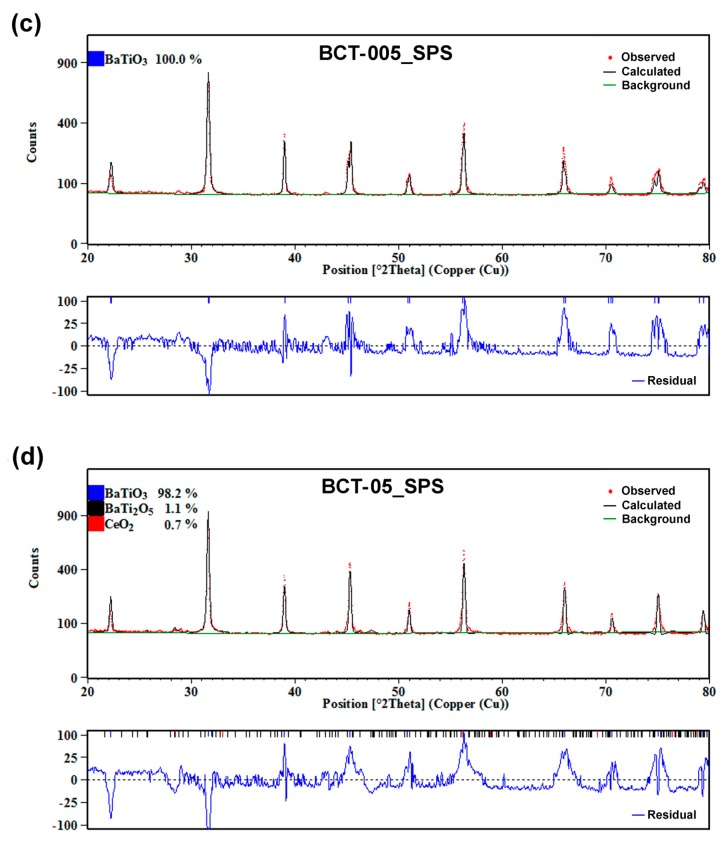
(**a**) XRD patterns recorded at room temperature for Ba_1−x_Ce_x_Ti_1−x/4_O_3_ ceramics obtained by spark plasma sintering (SPS) at 1050 °C/2 min, followed by post-sintering re-oxidation thermal treatment at 1000 °C/16 h; (**b**) details (light blue rectangle of Figure 6a) of the region corresponding to diffraction angles 2*θ* = 44.5–46.5°; (**c**,**d**) results of Rietveld analyses of X-ray diffraction data for the SP-sintered ceramics: (**c**) BCT-005_SPS and (**d**) BCT-05_SPS.

In the case of the ceramics obtained by spark plasma sintering, the corresponding XRD patterns also revealed a clear tendency towards single-phase compositions (Figure 6a). For the sample with higher cerium content (BCT-05_SPS), a small amount of CeO_2_ was identified at the detection limit, most likely because of incipient oxidation of Ce^3+^ to Ce^4+^ which takes place during the post-sintering thermal treatment. For this reason, the content of Ce^3+^ in the perovskite solid solution was slightly lower that that stipulated by the nominal formula. Consequently, a small amount of BaTi_2_O_5_ was also expelled from the perovskite lattice in order to preserve the stoichiometric ratio (Table 2 and Figure 6d). These data are in agreement with the results reported by Makovec and Kolar [50], who showed that after annealing at temperatures of 1000–1100 °C, internal partial oxidation of Ce^3+^ to Ce^4+^ accompanied by heterogeneous precipitation of small amounts of CeO_2_ and polytitanate phases occurs in the perovskite matrix.

For the ceramic specimen with low Ce^3+^ content (BCT-005_SPS), the oxidation process was negligible, so quantitative formation of the perovskite Ce^3+^-BaTiO_3_ solid solution as a unique phase was indicated by the Rietveld analysis (Table 2 and Figure 6c).

The details (light blue rectangle of Figure 6a) of the region corresponding to diffraction angles 2*θ* = 44.5°–46.5° revealed similar structural features to those in the case of the ceramics obtained by conventional sintering (Figure 6b). For the ceramic sample BCT-005_SPS, even if the splitting of the (200) reflection is less pronounced, the left-side asymmetry of the profile of the diffraction peak suggests a tetragonal structure but with a lower tetragonality degree than that corresponding to the conventionally sintered specimen of similar composition (BCT-005_CS).

In the case of the ceramic with high cerium content (BCT-05_SPS), the symmetric profile of the (200) reflection indicates a cubic structure (Figure 6b). These observations were sustained by the data provided by the Rietveld refinement (Table 2).

A reduction of the unit cell volume with increasing Ce^3+^ content was revealed for all the ceramic samples, no matter the sintering procedure. The slightly higher values of unit cell volume specific to the perovskite phase in the spark-plasma-sintered ceramics relative to the conventionally sintered specimens of similar composition could be explained in terms of higher inter-ionic distances induced by the presence of smaller grains, with lower crystallinity (Table 2).

#### 3.3.2. Microstructure

**Figure 7:**
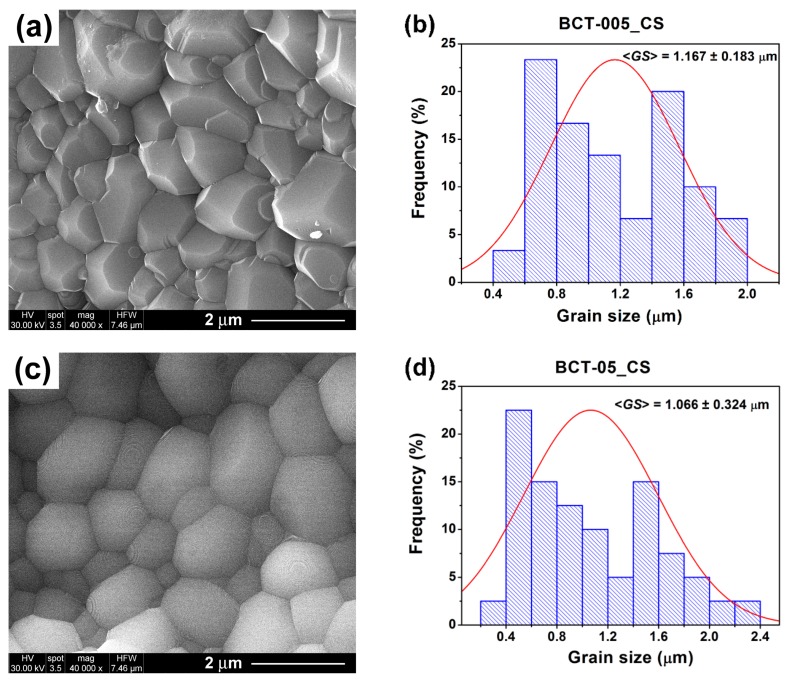
(**a**,**c**) FE-SEM images and (**b**,**d**) histograms indicating the grain size distribution for the conventionally sintered ceramics: (**a**,**b**) Ba_0.995_Ce_0.005_Ti_0.99875_O_3_ (BCT-005_CS) and (**c**,**d**) Ba_0.95_Ce_0.05_Ti_0.9875_O_3_ (BCT-05_CS).

The FE-SEM image of the ceramic sample with low cerium content obtained after conventional sintering at 1300 °C/4 h (BCT-005_CS) shows a fine-grained, homogeneous microstructure consisting of polyhedral, well-faceted grains (with truncated edges and corners) uniform in shape and size and with a small amount of intergranular porosity (Figure 7a). An average grain size <*GS*> of 1.167 µm (Table 2, Figure 7b), lower than the 5 µm reported in the case of sol-gel La^3+^-doped BaTiO_3_ ceramics of similar composition and sintered under similar conditions [10], was estimated in this case.

An increase in Ce^3+^ content only slightly affected the average grain size, but it seems to have promoted densification and induced a change in the morphology of the grains, which became rounded, without faces, edges, or corners. However, the grains exhibited well-defined boundaries and perfect triple junctions, while the intergranular pores were almost entirely missing, as the FE-SEM image in Figure 7c revealed. An average grain size of 1.066 µm was estimated for the specimen BCT-05_CS (Table 2, Figure 7d).

**Figure 8:**
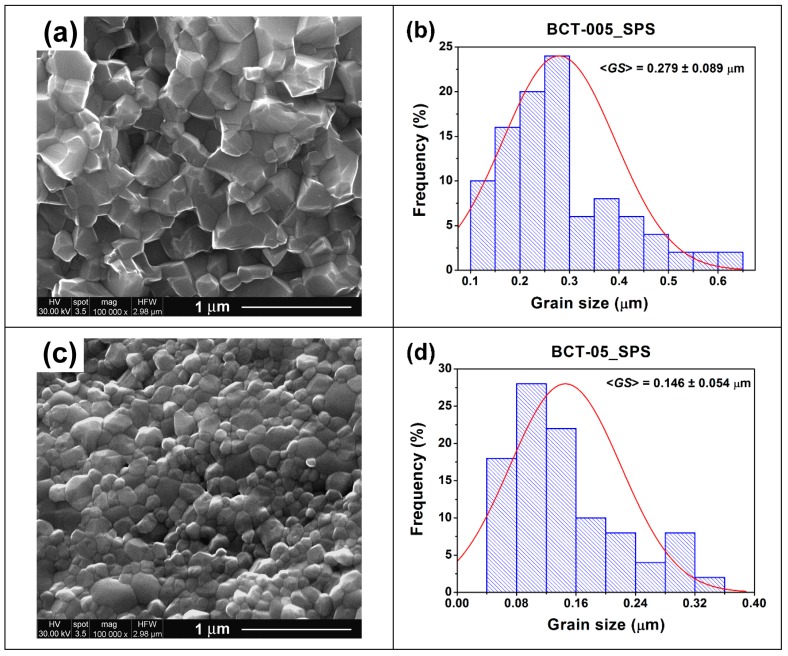
(**a**,**c**) FE-SEM images and (**b**,**d**) histograms indicating the grain size distribution for the spark-plasma-sintered ceramics: (**a**,**b**) Ba_0.995_Ce_0.005_Ti_0.99875_O_3_ (BCT-005_SPS) and (**c**,**d**) Ba_0.95_Ce_0.05_Ti_0.9875_O_3_ (BCT-05_SPS).

The ceramics obtained by spark plasma sintering exhibited denser microstructures composed of grains with significantly lower sizes but with similar morphologies relative to the samples resulting from conventional sintering (Figure 8a–c). Relative density values of 97.1% and 98.6% and average grain size values of 0.279 and 0.146 µm were determined for the ceramics denoted BCT-005_SPS and BCT-05_SPS, respectively (Table 2, Figure 8b,d). As in the case of the conventionally sintered sample, the SP-sintered specimen with low Ce^3+^ content (BCT-005_SPS) exhibited faceted polyhedral grains, tightly welded together, generating in some regions larger clusters composed of nanometric subgrains separated by dislocation networks (Figure 8a). An increase in cerium concentration not only induced a change in the grain morphology but also determined an obvious decrease in the average grain size. Thus, the pore-free specimen with high Ce^3+^ content (BCT-05_SPS) consisted of small, equiaxial grains and exhibited slightly higher densification and an almost unimodal grain size distribution (Figure 8c,d). FE-SEM investigations in back-scattered electrons (BSE) mode did not reveal the presence of secondary phases detected by XRD measurements, most likely because of their small amounts. It is worth mentioning that spark plasma sintering performed at 1050 °C for 2 min followed by re-oxidation thermal treatment at 1000 °C for 16 h induced an increase in the average grain size by 2.4–2.6 times compared to the average particle size of the starting sol-gel Ce^3+^-doped BaTiO_3_ powders. On the other hand, regarding the conventional sintering, a higher grain growth rate was estimated for the sample with higher cerium content (BCT-05_CS) than for BCT-005_CS, taking into account the values of the average particle sizes of the related powders.

#### 3.3.3. Dielectric and Ferroelectric Properties

**Figure 9:**
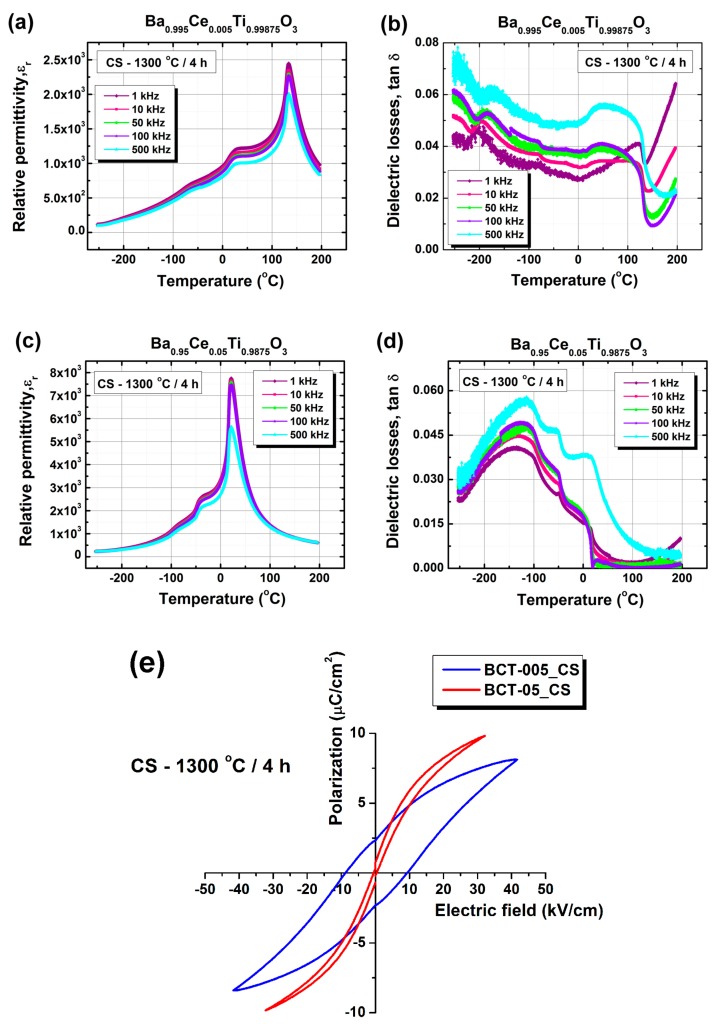
(**a**,**c**) Relative permittivity and (**b**,**d**) dielectric losses vs. temperature for the conventionally sintered ceramics described by the nominal formula Ba_1−x_Ce_x_Ti_1−x/4_O_3_: (**a**,**b**) BCT-005_CS; (**c**,**d**) BCT-05_CS. (**e**) *P–E* dependence for the Ce^3+^-doped BaTiO_3_ ceramics obtained by conventional sintering.

Figure 9a–d presents the temperature dependence of the dielectric properties recorded for five frequencies, in the range of 1 kHz–500 kHz, for the ceramics obtained by conventional sintering. Both samples showed a well-defined, sharp, and frequency-independent maximum of permittivity assigned to the ferroelectric–paraelectric phase transition at the Curie temperature (*T*_C_) (Figure 9a,c). A small frequency dispersion was observed only in the ferroelectric phase, mainly for the specimen with low cerium content (BCT-005_CS), whereas in the paraelectric state (above *T*_C_), the permittivity was almost frequency-independent for both samples, irrespective of the amount of Ce^3+^ solubilized in the perovskite lattice.

The samples showed good dielectric properties, with high values of relative permittivity and dielectric losses below 0.07 in the temperature range of −200 to +200 °C. The BaTiO_3_ sample doped with 5 atom % Ce^3+^ (BCT-05_CS) showed a drop of the loss tangent to below 0.01 at temperatures above the Curie temperature (Figure 9d). This insulating behavior is consistent with full electrical compensation by cation vacancies, homogeneously distributed inside the perovskite grains. These results are in agreement with the data reported earlier for ceramics with similar composition derived from powders synthesized using the modified Pechini method [35]. On the contrary, the BaTiO_3_ ceramic sample doped with a significantly lower Ce^3+^ content (0.5 atom %) exhibited an increase in the tangent loss, particularly at higher temperatures (above *T*_C_) and at lower frequencies (Figure 9b). Higher values of dielectric losses in the low frequency range are most likely associated with thermally activated space charge effects (Maxwell–Wagner phenomena), which commonly occur in lightly donor-doped BaTiO_3_ due to the grain boundary contribution or to the ionization of oxygen vacancies in the sintered ceramics [9,10,51].

For the lightly doped BCT-005_CS sample, the maximum permittivity at 1 kHz reached a value of 2448 at a Curie temperature of 133 °C, close to that specific to the undoped BaTiO_3_ ceramic, indicating that at room temperature this specimen was found in its ferroelectric state (Figure 9a and Table 3). The maximum permittivity was lower than that reported by Hwang and Han for their Ba_0.0995_Ce_0.005_TiO_3_ ceramic sample prepared by the Pechini method but sintered in air for 5 h at a higher temperature of 1380 °C [21]. The other two strongly flattened permittivity maxima recorded at *T*_1_ = 25 °C and *T*_2_ = −69 °C correspond to the tetragonal–orthorhombic (T–O) and orthorhombic–rhombohedral (O–R) phase transitions (Figure 9a). It must be noted that even though the best fit obtained by Rietveld analysis of the diffraction data indicated a tetragonal structure, the results of the dielectric measurements show that, actually, at room temperature, a mixture of tetragonal and orthorhombic modifications was found in the BCT-005_CS sample. The higher values of *T*_1_ and *T*_2_ than those corresponding to the undoped BaTiO_3_ single crystals (for which *T*_1_ = 5 °C and *T*_2_ = −90 °C) seem to be related to the small grain size of this polycrystalline ceramic sample.

The specimen with high Ce^3+^ addition (BCT-05_CS) showed a ferroelectric–paraelectric phase transition in the proximity of room temperature. As in the case of the BCT-005_CS ceramic, the sharp permittivity maximum suggests a first-order phase transition, specific to a typical ferroelectric material. A Curie temperature of 21 °C with a permittivity maximum of 7758 was determined in this case (Figure 9c and Table 3). This result is in agreement with the structural data, which showed cubic symmetry of the unit cell for the sample BCT-05_CS, proving that this ceramic was already found in the paraelectric state. The evolution of the Curie temperature with increasing donor dopant concentration shows that the Ce^3+^ solute was exclusively incorporated on Ba sites in the perovskite lattice. It has to be mentioned that for a ceramic sample with the same composition, derived from a powder prepared by the Pechini method and sintered in similar conditions, a slightly higher Curie temperature of 25 °C was reported in our previous work [35], which suggests that the wet chemical synthesis procedure of the starting oxide powder could play a certain role in stabilizing the paraelectric state at room temperature. A decreasing rate of −24.9 °C/atom % of Ce^3+^ was determined for the sol-gel ceramics described by the nominal formula Ba_1−x_Ce_x_Ti_1−x/4_O_3_ investigated in this study. This value is higher compared to the −18 °C/atom % reported in the literature by Jing et al. for Ba_1−x_Ce_x_Ti_1−x/4_O_3_ specimens prepared by the Pechini procedure [31].

A decreasing trend with increasing solute content, specific to *A*-site doping in BaTiO_3_, was also recorded for the T–O and O–R phase transition temperatures, which showed values of −44 and −93 °C, respectively (Table 3).

Measurements of *P*–*E* dependence were performed only for the ceramics obtained by conventional sintering. The higher leakage currents in spark-plasma-sintered ceramics did not allow us to measure the polarization versus the electric field, even for the single-phase specimen BCT-005_SPS.

In the case of the conventionally sintered sample with a small cerium addition, the hysteretic *P*–*E* dependence at fields ranging between −40 and +40 kV/cm clearly indicated the ferroelectric state. The sample labelled BCT-005_CS exhibited a tilted hysteresis loop, characterized by coercive fields of 8.7 kV/cm, saturation polarization of 8.1 µC/cm^2^, and a low remnant polarization value of 2.3 µC/cm^2^ (Figure 9e).

Regarding the specimen with high Ce^3+^ content (BCT-05_CS), the drop in coercivity and remnant polarization was consistent with the proximity of the paraelectric state at room temperature (Figure 9e). However, the “*S*” shape of the hysteresis loop seems to indicate a superparaelectric state, like that specific to the relaxor systems, rather than a typical paraelectric state, characterized by linear *P–E* dependence [52,53,54,55]. This suggests that at room temperature, a more complicated local structure, involving polar nano-regions consisting of unit cells with low tetragonality, embedded in a matrix of cubic unit cells, might be present in this sample. This kind of structure cannot be detected by XRD measurements, which involves averaging over at least 10,000 unit cells in the calculation of unit cell parameters.

**Figure 10:**
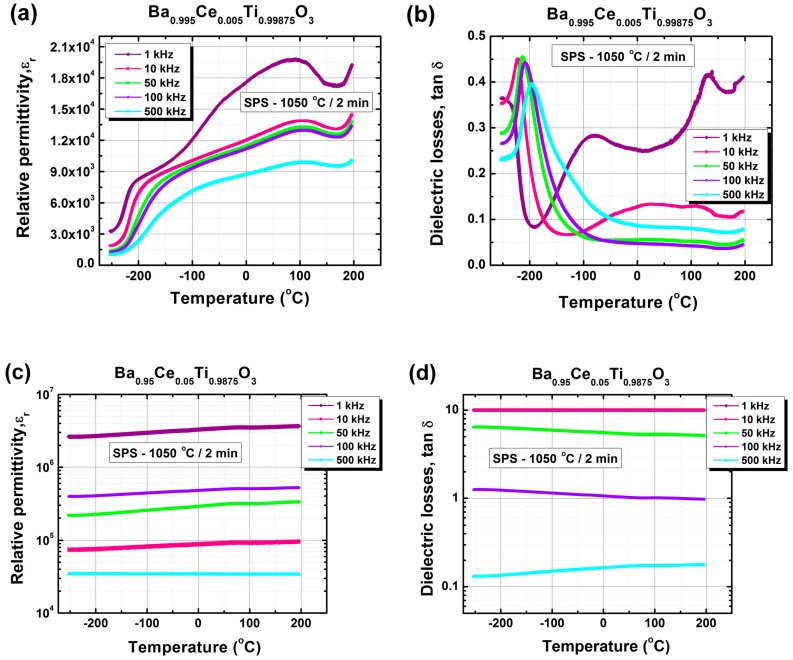

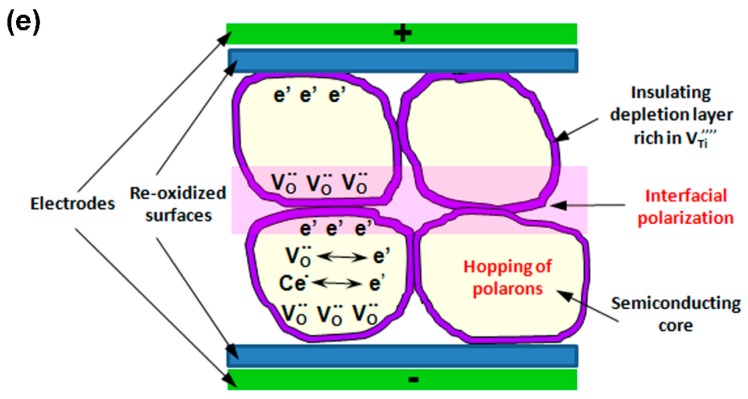
(**a**,**c**) Relative permittivity and (**b**,**d**) dielectric losses vs. temperature for the spark-plasma-sintered ceramics described by the nominal formula Ba_1−x_Ce_x_Ti_1−x/4_O_3_: (**a**,**b**) x = 0.005 (BCT-005_SPS); (**c**,**d**) x = 0.05 (BCT-05_SPS). (**e**) A schematic representation of the processes generating colossal permittivity in the BTC-05_SPS ceramic sample.

The spark-plasma-sintered ceramics with the nominal formula Ba_0.995_Ce_0.005_Ti_0.99875_O_3_ exhibited much higher permittivity values over the whole investigated temperature range when compared to the conventionally sintered samples with similar composition. The temperature dependence of relative permittivity was flattened, showing at 1 kHz a broad feature centered around the temperature of 91 °C (Figure 10a and Table 3). This might correspond to a diffuse ferroelectric–paraelectric phase transition caused by the low grain size, as reported in the case of undoped BaTiO_3_ ceramics consolidated by spark plasma sintering [56,57,58,59,60,61]. The T–O phase transition was barely detected as an asymmetric feature centered at −49 °C, while the shoulder at approximately −214 °C was assigned to the O–R phase transition. These data show a much more pronounced shift of the phase transition temperatures *T*_1_ and *T*_2_ towards lower temperature values compared to that specific to the Curie temperature, *T*_C_. Very high permittivity values of 800–20,000 were recorded at 1 kHz over the entire temperature range of −250 to +200 °C, and a maximum permittivity of 19,782 was found at 91 °C. Increasing frequency induced a strong flattening of the temperature dependence of the relative permittivity, so that at 500 kHz, only the ferroelectric–paraelectric phase transition could be identified, with the maximum permittivity reaching 9915 at 107 °C (Figure 10a). Regarding the dielectric losses, higher tan *δ* values of 0.27–0.42 and 0.07–0.13 were recorded in the temperature range of −100 to 200 °C for lower frequencies of 1 kHz and 10 kHz, respectively (Figure 10b, Table 3). At higher frequencies (of 50–500 kHz), in the same temperature range, the dielectric tangent exhibited values below 0.07, similar to those obtained for the conventionally sintered bulk ceramics with similar Ce^3+^ content. A strong anomaly in the temperature dependence of the dielectric loss tangent, the origin of which is still not clear but seems to be related to the (O–R) phase transition, was found in the low temperature range of −250 to +200 °C. This anomaly shows a certain frequency dispersion, reflected by the decrease and concurrent shift towards slightly higher temperature values of the tangent loss maximum with increasing frequency, as in the case of the relaxor systems [52,53] (Figure 10b). The higher values of dielectric losses in the low frequency region in spark-plasma-sintered BaTiO_3_ ceramics, relative to those in conventionally sintered samples, originated from the higher leakage currents induced by the higher density of interface states associated with the lower grain size. Besides this, one cannot exclude the presence of a very small concentration of oxygen vacancies which can persist even after prolonged re-oxidation thermal treatments; these might induce a conductive contribution via a mechanism involving small polarons hopping [62]. However, the white color in the cross section of the sample indicates that the insulating behavior prevails.

The sample with high cerium content, described by the nominal formula Ba_0.95_Ce_0.05_Ti_0.9875_O_3_, showed colossal values of effective permittivity, almost temperature independent, which decreased from 3 × 10^6^ to 2 × 10^4^ when the frequency increased from 1 kHz to 500 kHz (Figure 10c). The same temperature invariance was recorded for dielectric losses, which decreased from tan *δ* >> 1 to tan *δ* = 0.2 when the frequency increased from 1 kHz to 500 kHz (Figure 10d). Such large values of effective permittivity and dielectric losses can be explained in terms of Maxwell–Wagner phenomena and rather indicate a semiconducting behavior. Similar characteristics were also reported for a fine-grained Ba_0.95_La_0.05_TiO_3_ ceramic sample prepared from nano-sized powders and consolidated by spark plasma sintering, where the colossal permittivity was found to arise from a conjugated effect of the re-oxidized insulating surface and high interfacial polarization inside the ceramic specimen [63,64]. This large interfacial polarization seems to be determined by a “brick-wall”-type electric microstructure due to the presence of highly semiconducting grain cores, with a high concentration of carriers (polarons) induced by both reducing conditions during the SPS process and a high concentration of donor dopant in association with the high density of very thin grain boundary depletion layers, rich in electron traps (cation vacancies) and consequently exhibiting insulating properties (Figure 10e). In a highly donor-doped, fine-grained barium titanate sample of grey color such as this, the semiconducting behavior prevails and can be explained in terms of a combination of the two alternative models proposed earlier by Takeuchi et al. to explain the presence of the conductive contribution induced by the oxygen deficiency in partially reduced undoped BaTiO_3_ ceramics [39].

## 4. Conclusions

Ce^3+^-doped BaTiO_3_ powders described by the nominal formula Ba_1−x_Ce_x_Ti_1−x/4_O_3_ with x = 0.005 and 0.05 were synthesized by the acetate variant of the sol-gel method. The structural parameters, particle size, and morphology are strongly dependent on the Ce^3+^ content. From these powders, ceramics were prepared by conventional sintering at 1300 °C/4 h or by spark plasma sintering at 1050 °C/2 min. For all the investigated ceramics, irrespective of their composition and sintering procedure, the solute was exclusively incorporated as Ce^3+^ on Ba^2+^ sites, acting as a donor dopant. The results of the dielectric and *P*–*E* measurements were in good agreement with the values of the structural parameters calculated based on the XRD data, revealing that at room temperature, the ceramic with low cerium content (x = 0.005) was in the ferroelectric state, while the sample with a significantly higher Ce^3+^ concentration (x = 0.05) was found to be in the proximity of the ferroelectric–paraelectric phase transition. Curie temperatures of 133 °C and 21 °C and values of maximum permittivity of 2448 and 7758 were determined for the conventionally sintered ceramics with x = 0.005 and x = 0.05, respectively. The fine-grained and dense ceramic sample with low solute content resulting from spark plasma sintering exhibited insulating behavior at 1 kHz, with significantly higher values of relative permittivity (*ε*_r_ ~20,000) and dielectric losses (tan *δ* = 0.2–0.4) over the entire investigated temperature range relative to the conventionally sintered sample with similar composition. The spark-plasma-sintered Ce^3+^-BaTiO_3_ specimen with high cerium content (x = 0.05) showed a fine-grained microstructure and an almost temperature-independent colossal dielectric constant (*ε*_r_ ~ 3.35 × 10^6^). However, in this case, the enormous dielectric losses rather indicated a semiconducting behavior.

To conclude, in this work we revealed that by reducing the grain size from micro- toward nano-scale, the interplay of the influences of both the composition and the microstructural features determined opposing evolution in the two BCT ceramics. Thus, a dielectric of average performance (the BCT ceramic sample with x = 0.005) acquired significantly higher permittivity, more stable with temperature, while a better dielectric (the BCT sample with x = 0.05) was converted into a typical semiconductor, due to the high concentration of polarons.

## Figures and Tables

**Table 1:** Phase composition and structural parameters obtained by Rietveld refinement and average particle size estimated from TEM investigations for the sol-gel Ce^3+^-doped BaTiO_3_ powders annealed at 900 °C/2 h.

Formula		Ba_0.995_Ce_0.005_Ti_0.99875_O_3_	Ba_0.95_Ce_0.05_Ti_0.9875_O_3_
Sample Symbol		BCT-005	BCT-05
Phase composition		BCTss-100%	BCTss-100%
BCTss structure		Tetragonal, P4mm	Cubic, Pm3m
Unit cell parameters	*a* (Å)	4.003105 ± 0.000113	4.001052 ± 0.001342
	*b* (Å)	4.003105 ± 0.000113	4.001052 ± 0.001342
	*c* (Å)	4.016862 ± 0.000156	4.001052 ± 0.001342
Tetragonality, *c*/*a*		1.0034	1.0000
Unit cell volume, *V* (Å^3^)		64.36961	64.05051
Theoretical density, *ρ*_t_ (g/cm^3^)		6.014	6.032
*R* profile, *R_p_*		5.04481	4.89943
Weighted *R* profile, *R_wp_*		6.56691	6.38165
Goodness of fit, χ^2^		0.01703	0.01539
Crystallite size, <*D*> (nm)		33.66 ± 10.03	25.98 ± 5.35
Internal strains, <*S*> (%)		0.27 ± 0.05	0.35 ± 0.07
Particle size, <*d*_TEM_> (nm)		109.25 ± 30.68	60.93 ± 13.71

BCTss, Ce^3+^-BaTiO_3_ solid solution: Tetragonal, P4mm (ICDD card no. 01-081-8524); Cubic, Pm3m (ICDD card no. 01-083-3859).

**Table 2:** Structural/microstructural parameters of Ce^3+^-doped BaTiO_3_ ceramics derived from sol-gel powders and consolidated by conventional sintering or spark plasma sintering.

Formula		Ba_0.995_Ce_0.005_Ti_0.99875_O_3_		Ba_0.95_Ce_0.05_Ti_0.9875_O_3_	
Sample Symbol		BCT-005_CS	BCT-005_SPS	BCT-05_CS	BCT-05_SPS
Sintering procedure/conditions		CS 1300 °C/4 h	SPS 1050 °C/2 min	CS 1300 °C/4 h	SPS 1050 °C/2 min
Phase composition		BCTss-100%	BCTss-100%	BCTss-100%	BCTss-98.2% BT_2_-1.1% C-0.7%
BCTss structure		Tetragonal, P4mm	Tetragonal, P4mm	Cubic, Pm3m	Cubic, Pm3m
Unit cell parameters	*a* (Å)	3.994169 ± 0.000108	4.000073 ± 0.000273	3.999053 ± 0.000054	3.999968 ± 0.000167
	*b* (Å)	3.994169 ± 0.000108	4.000073 ± 0.000273	3.999053 ± 0.000054	3.999968 ± 0.000167
	*c* (Å)	4.024993 ± 0.000127	4.021890 ± 0.000344	3.999053 ± 0.000054	3.999968 ± 0.000167
Tetragonality, *c*/*a*		1.0077	1.0054	1.0000	1.0000
Unit cell volume, *V* (Å^3^)		64.21228	64.35261	63.95455	63.99847
Theoretical density, _t_ (g/cm^3^)		6.027	6.014	6.041	6.037
*R* profile, *R*_p_		6.48709	6.75345	7.53511	7.3148
Weighted *R* profile, *R_wp_*		9.1197	10.6243	10.8429	10.79943
Goodness of fit, χ^2^		0.47706	0.45168	0.62327	0.48938
Crystallite size, <*D*> (nm)		48.72 ± 4.31	45.85 ± 6.80	171.43 ± 7.49	45.05 ± 6.37
Internal strains, <*S*> (%)		0.19 ± 0.07	0.21 ± 0.12	0.18 ± 0.08	0.22 ± 0.11
Relative density, *ρ*_r_ (%)		91.7	95.8	97.1	98.6
Grain size, <*GS* > (µm)		1.167 ± 0.183	0.279 ± 0.089	1.066 ± 0.324	0.146 ± 0.054

*CS*, conventional sintering; *SPS*, spark plasma sintering; BCTss, Ce^3+^-BaTiO_3_ solid solution: Tetragonal, P4mm (ICDD card no. 01-081-8524); Cubic, Pm3m (ICDD card no. 01-083-3859); BT_2_, BaTi_2_O_5_: Monoclinic, C2 (ICDD card no. 04-012-4418); C, CeO_2_: Cubic, Fm3m (ICDD card no. 00-067-0121).

**Table 3:** Dielectric properties at 1 kHz for the Ce^3+^-doped BaTiO_3_ ceramics derived from sol-gel powders and consolidated by conventional sintering or spark plasma sintering.

Formula	Ba_0.995_Ce_0.005_Ti_0.99875_O_3_		Ba_0.95_Ce_0.05_Ti_0.9875_O_3_	
Sample Symbol	BCT-005_CS	BCT-005_SPS	BCT-05_CS	BCT-05_SPS
Sintering procedure/conditions	CS 1300 °C/4 h	SPS 1050 °C/2 min	CS 1300 °C/4 h	SPS 1050 °C/2 min
*ε′*_max_	2448	19782	7758	3.67 × 10^6^
*T*_C_	133	91	21	−
*T*_1_	25	−49	−44	−
*T*_2_	−69	−214	−93	−
*ε′*_RT_	1163	18367	7695	3.35 × 10^6^
tan *δ*_RT_	0.0287	0.2498	0.0098	~10
